# Transitioning from human primordial germ cells to embryonic germ cells

**DOI:** 10.1016/j.stemcr.2025.102749

**Published:** 2025-12-26

**Authors:** Kenyu Iwatsuki, Yasuhiro Takashima

**Affiliations:** 1 Department of Life Science Frontiers, CiRA, Kyoto University, 53 Kawahara-cho, Shogoin, Sakyo-ku, Kyoto 606-8507, Japan

## Abstract

Primordial germ cells (PGCs) can give rise to pluripotent embryonic germ cells (EGCs) in rodents. Leitch and colleagues in this issue successfully derived human EGC-like cells from PGC-like cells and applied comprehensive multi-omic profiling to resolve their pluripotent and epigenetic dynamics, providing new insights into potential mechanisms underlying human germ cell tumorigenesis.

## Teaser

Primordial germ cells (PGCs) can give rise to pluripotent embryonic germ cells (EGCs) in rodents. Stucchi et al. in this issue successfully derived human EGC-like cells from PGC-like cells and applied comprehensive multi-omic profiling to resolve their pluripotent and epigenetic dynamics, providing new insights into potential mechanisms underlying human germ cell tumorigenesis.

## Main text

Understanding how human germ cells regain pluripotency is essential for clarifying the origin of germ cell tumors (GCTs). In early post-implantation embryos, primordial germ cells (PGCs) arise as a small cluster and ultimately give rise to mature gametes—oocytes or spermatozoa—that enable the transmission of genetic information to the next generation (Saitou and Miyauchi, 2016). In mice, BMP4 signals from the extraembryonic ectoderm specify the post-implantation epiblast toward the PGC lineage, triggering reacquisition of pluripotency followed by genome-wide epigenetic reprogramming during migration to the gonads. Notably, these two processes—germ cell fate specification and extensive epigenetic reprogramming—are conserved hallmarks of PGC development (Saitou and Miyauchi, 2016). Disruptions in these steps can lead to the formation of GCTs (Nicholls and Page, 2021). However, the mechanisms by which only a subset of PGCs initiate tumorigenesis remain unresolved. Of particular interest, mouse PGCs readily give rise to embryonic germ cells (EGCs) *in vitro*, which display self-renewal and pluripotent characteristics (Matsui et al., 1992), similar to both pluripotent stem cells (PSCs) and GCTs. Rodent naive EGCs can contribute to chimeras (Matsui et al., 1992; Leitch et al., 2010), demonstrating full pluripotency. Human PGCs have also been used to derive EGCs, but human EGCs (hEGCs) generated to date require feeder cells, serum, and additional growth factors (Turnpenny et al., 2006). Although these hEGCs show self-renewal, they fail to form teratomas, indicating incomplete pluripotency. It has been suggested that differences between mouse and human EGCs may reflect the developmental stage of the starting PGCs. Due to ethical limitations, only late-stage human PGCs (weeks 6–9 post conception) that have reached the gonadal ridge can be used, thus hindering the establishment of human EGCs that fully recapitulate PSC-like pluripotency.

PGC-like cells (PGCLCs) generated from PSCs resemble early-stage human PGCs (Saitou and Miyauchi, 2016) and serve as an ethically accessible alternative to migrating PGCs. Multiple induction protocols have been developed, with the current standard involving the differentiation of primed human PSCs (hPSCs) to pre-mesendoderm or incipient mesoderm-like cells (iMeLCs), followed by aggregation in BMP2/4-based media. Recent advances demonstrated that human PGCLCs (hPGCLCs) can be maintained long term on feeder cells and give rise to EGC-like cells (EGCLCs) under these conditions (Kobayashi et al., 2022) or progress beyond the PGC stage (Murase et al., 2024). These findings suggest that hPGCLCs retain germline-like properties yet possess latent potential to re-acquire pluripotency. Nevertheless, the detailed molecular processes—transcriptomic and epigenomic—that accompany the transition from hPGCLCs to EGCLCs remain incompletely understood.

**Figure 1:**
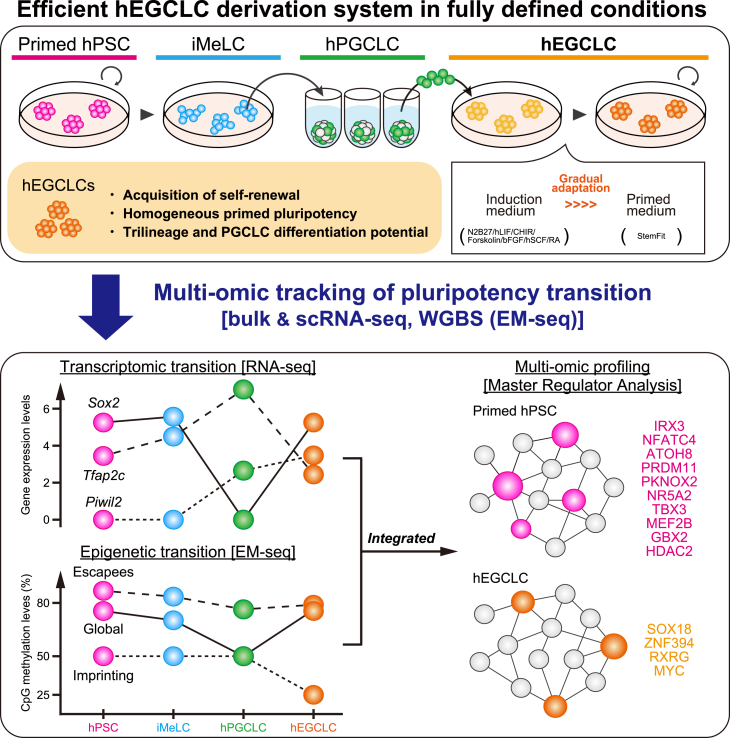
Derivation of hEGCLCs and multi-omic profiling across sequential stages Experimental workflow illustrating the efficient derivation of hEGCLCs from sorted hPGCLCs. A gradual transition from the induction medium to the StemFit-based primed hPSC maintenance medium is a critical step for establishing stable hEGCLCs. Comprehensive RNA-seq and WGBS analyses across hPSCs, iMeLCs, hPGCLCs, and hEGCLCs provide detailed transcriptomic and epigenetic landscapes, enabling the identification of key regulatory transcription factors associated with each developmental stage. Magenta, blue, green, and orange represent primed PSCs, iMeLCs, hPGCLCs, and hEGCLCs, respectively.

In this issue of *Stem Cell Reports*, Stucchi et al., 2025 report the successful derivation of human EGCLCs (hEGCLCs) from day 4 or day 6 hPGCLCs under fully defined, serum- and feeder-free conditions (Stucchi et al., 2025). hPGCLCs were first cultured for 2 days in a hEGCLC induction medium, N2B27 supplemented with hLIF, GSK3 inhibitor CHIR99021, forskolin, bFGF, hSCF, and retinoic acid, followed by gradual adaptation to StemFit medium, which supports primed pluripotency (Figure 1). Under these conditions, 3%–18% of hPGCLCs converted into hEGCLCs within 2 weeks. Importantly, these induction conditions parallel those used for mouse EGCLC derivation, suggesting that molecular triggers of the PGC-to-EGC conversion may be conserved across mammals. However, it is noteworthy that the maintenance phase in this study relied on primed PSC culture medium rather than naive conditions. Naive human PSCs, which correspond to the pre-implantation epiblast, represent a pluripotent state distinct from primed PSCs, which correspond to the post-implantation epiblast (Takashima et al., 2014). This distinction highlights that, although the induction step resembles the mouse system, the subsequent stabilization of hEGCLCs is achieved under primed, not naive, pluripotency conditions. Whether hEGCLCs can be induced under naive conditions in humans remains an open question.

Live imaging using BTAG (BLIMP1-tdTomato/TFAP2C-EGFP) and SOX2 reporters revealed that the transition from hPGCLCs to hEGCLCs involves the downregulation of PGC-specific genes and reciprocal activation of pluripotency-associated genes. The resulting hEGCLCs exhibited self-renewal, trilineage differentiation, and the capacity to re-induce hPGCLCs, consistent with previous findings (Kobayashi et al., 2022).

To investigate the molecular mechanisms underlying this transition, the authors performed RNA sequencing (RNA-seq) and whole-genome bisulfite sequencing (WGBS) across sequential stages—from hPSCs to iMeLCs, hPGCLCs, and EGCLCs (Figure 1). When comparing primed hiPSCs with hEGCLCs, bulk RNA-seq identified 467 differentially expressed genes (DEGs) at passage 0 but only ∼50 DEGs at passages 3 and 5, indicating early acquisition of a transcriptional state closely resembling primed pluripotency. Single-cell RNA-seq confirmed that hEGCLCs form a homogeneous population transcriptionally similar to hiPSCs. Yet, consistent with previous reports (Kobayashi et al., 2022), certain PGC-specific genes, including *PIWIL2*, remained partially expressed during the transition. *UHRF1*, a regulator of DNA methylation maintenance and a gene implicated in tumorigenesis (Yamaguchi et al., 2024), is especially noteworthy, as *UHRF1* expression increased during PGCLC induction and remained high in hEGCLCs. Understanding its function may yield important insights into latent pluripotency and GCT initiation.

WGBS revealed that global DNA methylation levels in hEGCLCs largely recovered to levels seen in hiPSCs. However, several imprint control regions, including *PLAG1*-*HYMAI* and *GNAS_XL*, exhibited reduced methylation—indicative of loss of imprinting (LOI). LOI is known to occur in naive PSCs (Paster et al., 2016), and its emergence during the hPGCLC-to-hEGCLC transition provides key insight into mechanisms driving abnormal demethylation. Escapee regions, which are resistant to reprogramming in PGCs and may contribute to transgenerational epigenetic inheritance (Saitou and Miyauchi, 2016), also showed distinct methylation dynamics: early-passage hEGCLCs retained profiles resembling hPGCLCs, whereas prolonged passaging shifted their methylation patterns toward those of hiPSCs.

To uncover key regulators of pluripotency-germline transitions, the authors performed master regulator analysis integrating transcriptomic and methylation datasets (Figure 1). While gene regulatory network analysis suggested that hiPSCs and hEGCLCs share similar core regulatory frameworks with common key regulators such as MYC, HDAC2, ZIC5, and SP3, master regulator analysis highlighted distinct cell-type-specific key regulators, identifying 10 primed hPSC-specific transcription factors (IRX3, NFATC4, ATOH8, PRDM11, PKNOX2, NR5A2, TBX3, MEF2B, GBX2, and HDAC2) and 4 hEGCLC-specific transcription factors (SOX18, ZNF394, RXRG, and MYC). These findings illustrate the value of multi-omic integration for dissecting pluripotent state transitions via PGCLCs.

## Declaration of interests

The authors declare no competing interests.
